# Acquired RUFY1-RET rearrangement as a mechanism of resistance to lorlatinib in a patient with CD74-ROS1 rearranged non-small cell lung cancer: A case report

**DOI:** 10.18632/oncotarget.28682

**Published:** 2025-02-05

**Authors:** Jenny L. Wu, Wade T. Iams

**Affiliations:** ^1^Vanderbilt University School of Medicine, Nashville, TN 37232, USA; ^2^Vanderbilt-Ingram Cancer Center, Nashville, TN 37232, USA

**Keywords:** ROS1 rearrangement, RET rearrangement, non-small cell lung cancer, targeted therapy, case report

## Abstract

*ROS1* and *RET* fusions are targetable mutations that occur in a subset of patients with non-small cell lung cancer (NSCLC). *ROS1* and *RET* have been understood to be independent oncogenic drivers which do not co-occur with other common tyrosine kinase receptor mutations except in the acquired resistance setting. Here we present a case of a patient with stage IV *CD-74-ROS1* fusion NSCLC discovered initially with RNA next generation sequencing (NGS) who acquired resistance to lorlatinib after 6 months on therapy through a novel *RUFY1-RET* fusion, detected only through RNA NGS. Combination therapy targeting RET and ROS1 using pralsetinib and lorlatinib achieved a partial response with limited durability of only four months. This is the first reported case of a *RET* fusion as a potential mechanism of resistance to lorlatinib, it identifies a novel *RET* fusion partner, and it emphasizes the importance of testing for acquired resistance mutations with both DNA and RNA at the time of progression in patients with targetable oncogenic drivers.

## INTRODUCTION

Among current molecular targets for advanced non-small cell lung cancer (NSCLC) are c-Ros oncogene 1 (*ROS1*) and rearranged during transfection (*RET*), which code for tyrosine kinase receptors. When these genes are rearranged with different fusion partners, they produce constitutively active proteins which drive oncogenesis [1]. *ROS1* and *RET* fusions have a similar prevalence of approximately 1–2% of NSCLC cases [2]. Notably, *ROS1* and *RET* fusions are hypothesized to act as independent oncogenic drivers and do not co-express *de novo* with other oncogenic mutations in *EGFR*, *ALK*, and *KRAS* [2, 3].

We present the case of a patient with stage IV *ROS1*-rearranged NSCLC who progressed on lorlatinib treatment after 6 months of disease control. A novel *RUFY1-RET* fusion concomitant with the patient’s original *ROS1* fusion was identified by RNA NGS on a biopsy of a progressing site. We hypothesize that the *RET* fusion developed as a mechanism of resistance to therapeutic targeting of the ROS1 fusion protein with lorlatinib. The patient then tolerated and initially responded to combined lorlatinib and pralsetinib. To our knowledge, this is the first report of an acquired *RET* fusion in a patient treated with lorlatinib, it identifies a novel *RET* fusion partner, emphasizes the importance of RNA NGS to identify oncogenic fusions, and it notes the first modestly successful dual lorlatinib and pralsetinib treatment for a heavily pretreated patient in this rare scenario.

## CASE PRESENTATION

**Figure 1:**
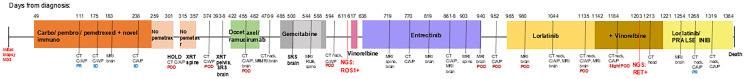
Treatment timeline with days from diagnosis. Abbreviations: PR: partial response; SD: stable disease; POD: progression of disease. Terms designated according to the RECIST criteria. Abbreviations: CT: computed tomography; C/A/P: chest, abdomen, pelvis; MRI: magnetic resonance imaging; XRT: radiation therapy.

A 42-year-old man who had never smoked presented with cough and wheezing (Figure 1, day 0 on the treatment timeline). A computed tomography (CT) scan of the chest showed a hilar mass, a mass in the lung, a small pleural effusion, and necrotic mediastinal lymphadenopathy. A TTF-1 positive adenocarcinoma of the lung with PD-L1 expression of 80% was diagnosed. A 324-gene Foundation One^®^ DNA NGS test at the time of diagnosis revealed *JUN* E108Q and *PARK2* P113fs*51 mutations and tumor mutation burden (TMB) of 9 mutations/Mb. The patient was started on carboplatin, pemetrexed, pembrolizumab, and a novel immunotherapy on a clinical trial with initial partial response and a duration of response of 10 months. At the time of initial progression, he was treated with docetaxel plus ramucirumab, and unfortunately progressed within 2 months. He was then treated with gemcitabine with disease control for 4 months.

At the time of progression on gemcitabine, a biopsy of an enlarging supraclavicular lymph node was sent for DNA and RNA NGS testing using a 648-gene Tempus xT^®^ assay. The results of the DNA NGS showed *CDKN2A*, *CDKN2B*, and *MTAP* loss with a TMB of 1.6 mutations/Mb. The RNA NGS revealed a *CD74-ROS1* chromosomal rearrangement; the allelic fraction of this fusion partner is unknown. With the discovery of the *ROS1*-rearrangement, entrectinib was initiated. The patient responded, though he experienced oligoprogression after 6 months and was treated with local radiation therapy. Entrectinib treatment continued for 10 months, but unfortunately a new lesion of the left thalamus was identified and growth of a cervical spinal lesion occurred; further imaging revealed progression of disease in the right lung and left adrenal gland and worsening upper abdominal and supraclavicular lymphadenopathy.

Following multiple sites of progression with CNS involvement on entrectinib, the patient was treated with lorlatinib per the National Comprehensive Cancer Network^®^ (NCCN^®^) guidelines [4]. After 6 months of disease control on lorlatinib, progression occurred in a cervical lymph node and retrosternal mass, though the CNS disease remained stable. Vinorelbine was added in combination with lorlatinib. Repeat DNA and RNA NGS testing from a biopsy of the progressing cervical lymph node using a Tempus xT^®^ assay revealed the persistent *CD74-ROS1* fusion, *LRP1B* copy loss, and TMB 1.6 mutations/Mb on DNA NGS and a *RUFY1-RET* rearrangement on RNA NGS.

**Figure 2:**
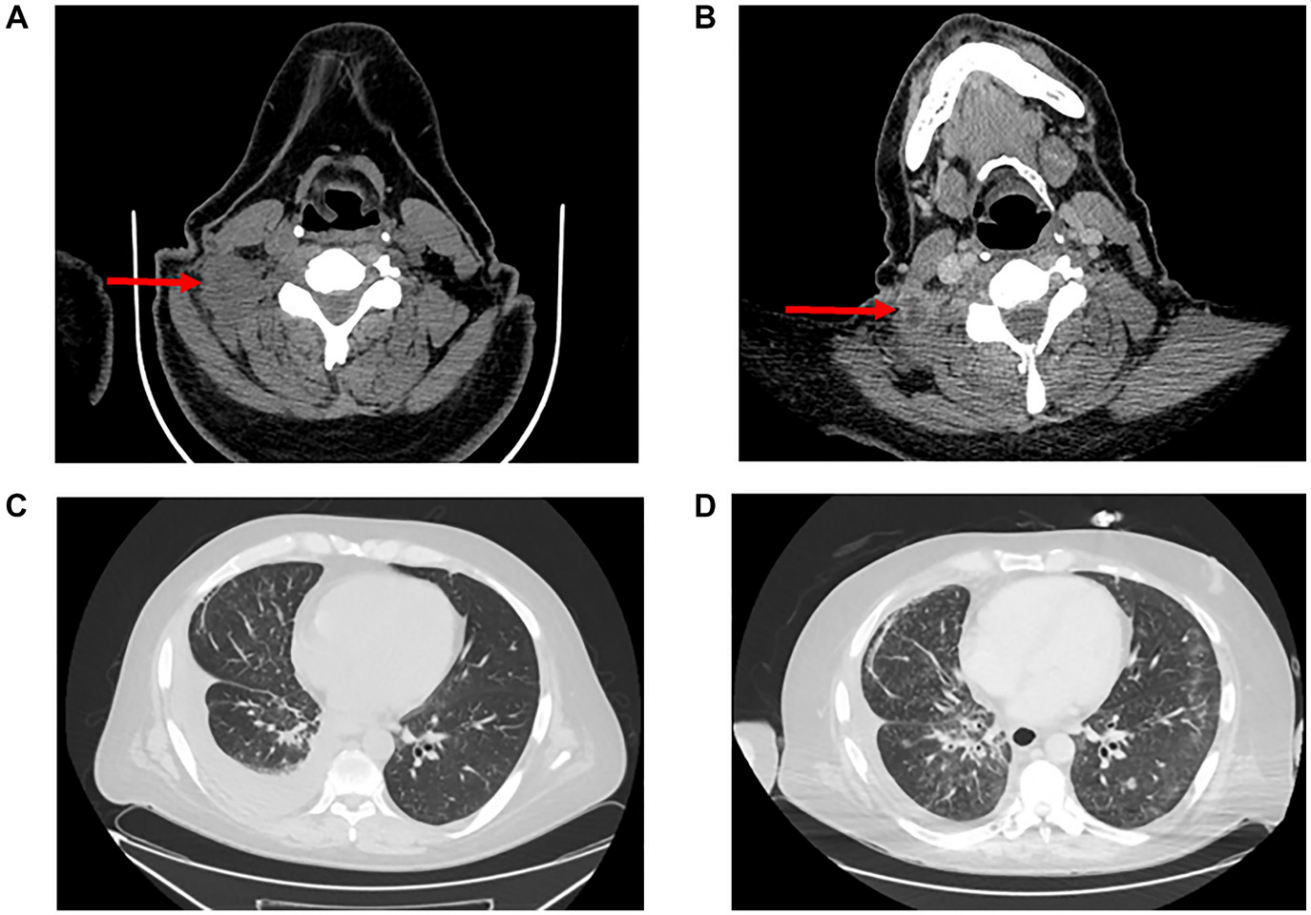
Radiographic response imaging to pralsetinib plus lorlatinib. (**A**) Computed tomography (CT) during lorlatinib treatment revealing right cervical lymph node. (**B**) CT showing decrease in lymph node size with necrosis after addition of pralsetinib. (**C**) Chest CT during lorlatinib treatment showing right-sided pleural effusion. (**D**) Chest CT showing decrease in effusion after pralsetinib treatment. Images A and C were taken on day 1135 post-diagnosis. Images B and D were taken on day 1268.

Pralsetinib (400 mg) was added in combination with dose reduced lorlatinib (from 100 mg to 75 mg), and vinorelbine was stopped. The patient had no severe adverse events, and a radiographic response was achieved on CT imaging one month later (Figure 2), with significant decrease in the right cervical lymph node, stable supraclavicular nodes, stable CNS and osseous metastases, and decrease in pulmonary disease and pleural effusion. Two months later, the noted lymph nodes remained stable on repeat imaging. Unfortunately, the patient died suddenly from respiratory failure after 4 months on pralsetinib plus lorlatinib due to disease progression.

## DISCUSSION

The case described highlights several critical and novel components of contemporary advanced NSCLC care. First, studies have shown that RNA NGS is more sensitive for the detection of chromosomal fusions compared to DNA NGS; unlike DNA sequencing, it is not impacted when fusion events involve long or repetitive intronic sequences [5]. The sensitivity of RNA NGS was demonstrated at the patient’s initial repeat biopsy demonstrating a *ROS1* fusion only on RNA NGS as well as the detection of the *RUFY1-RET* fusion on RNA NGS only at the time of resistance to lorlatinib. Therefore, RNA NGS should be included in all NSCLC tumor sequencing platforms.

The RNA NGS test at time of progression on gemcitabine revealed *ROS1* fusion, which prompted the initiation of entrectinib. Notably, the patient had a progression-free survival (PFS) on entrectinib of only 10 months, shorter than the PFS of 15.7 months noted in clinical trials in ROS1 TKI-naïve patients [6]. This could be attributed to the later-line use of entrectinib on this patient.

Acquired resistance to oncogene directed therapy can occur through on-target or off-target (bypass signaling) pathways. There is an emerging body of literature describing resistance to lorlatinib through bypass signaling [7]. While acquired *RET* fusions as a mechanism of resistance to EGFR TKIs have been increasingly documented in the literature (Table 1), this is the first reported case to our knowledge of an acquired activating *RET* fusion as a potential mechanism of resistance to lorlatinib.

**Table 1:** Studies of acquired *RET* fusions as resistance to EGFR TKIs in patients with NSCLC

Study	Study design	Baseline activating mutation	RET fusion partner	Resistance to treatment	Secondary treatment
Rotow et al. 2023 [8]	Prospective cohort study	EGFR	CCDC6-RET, NCOA4-RET	Osimertinib	Selpercatinib plus osimertinib
Wang et al. 2022 [9]	Retrospective cohort study	EGFR	KIF5B-RET, CCDC6-RET	3rd gen EGFR-TKIs	N/A
Piotrowska et al. 2018 [10]	Retrospective cohort study + case report	EGFR	CCDC6-RET, NCOA4-RET	Osimertinib	Pralsetinib plus osimertinib
Romagnolo et al. 2023 [11]	Case report	EGFR	NCOA-RET	Osimertinib	Pralsetinib plus osimertinib
Zhao et al. 2022 [12]	Case report	EGFR	CCDC6-RET	Osimertinib	Pralsetinib plus osimertinib

Analogous to the recent EGFR + RET inhibitor approach in the expanded access study [8], the patient was treated with lorlatinib and the RET inhibitor pralsetinib. Lorlatinib was dose-reduced from 100mg to 75 mg to assure tolerance of the combination. The patient did not experience any severe adverse events, and may have been able to tolerate a full dose combination. While imaging revealed an initial response, it was not durable, and the patient experienced terminal progression in 4 months. It is possible that the unique 5′ *RET* fusion partner (*RUFY1*) affected therapeutic durability. One patient with a *RUFY2-RET* fusion was reported in the recent expanded access selpercatinib plus osimertinib study, with that individual experiencing disease progression within 2 months [8].

In conclusion, we present a case of a patient with advanced *CD74-ROS1* fusion NSCLC who acquired resistance to lorlatinib concurrent with developing a novel *RUFY1-RET* fusion, with both oncogenic fusion events identified on RNA NGS only. Combination therapy using pralsetinib and lorlatinib was tolerable and achieved an initial response with four months of disease control. This case adds to the literature on bypass signaling as a mechanism of resistance to lorlatinib, providing evidence for *RET* activation as a novel escape mechanism and documenting tolerability and modest therapeutic efficacy with combination lorlatinib and pralsetinib in this rare scenario.
